# A network-pathway based module identification for predicting the prognosis of ovarian cancer patients

**DOI:** 10.1186/s13048-016-0285-0

**Published:** 2016-11-02

**Authors:** Xin Wang, Shan-shan Wang, Lin Zhou, Li Yu, Lan-mei Zhang

**Affiliations:** 1 Department of Gynaecology and Obstetrics, The 306 Hospital of PLA, Beijing, 100037 China; 2 Outpatient Pharmacy, Outpatient Department, NO.16 Chengzhuang Fengtai Distinct, Beijing, 100071 China

**Keywords:** Ovarian cancer, Reactome functional interactions, Markov clustering, Supervised principal components, Prognosis

## Abstract

**Background:**

This study aimed to screen multiple genes biomarkers based on gene expression data for predicting the survival of ovarian cancer patients.

**Methods:**

Two microarray data of ovarian cancer samples were collected from The Cancer Genome Atlas (TCGA) database. The data in the training set were used to construct Reactome functional interactions network, which then underwent Markov clustering, supervised principal components, Cox proportional hazard model to screen significantly prognosis related modules. The distinguishing ability of each module for survival was further evaluated by the testing set. Gene Ontology (GO) functional and pathway annotations were performed to identify the roles of genes in each module for ovarian cancer.

**Results:**

The network based approach identified two 7-gene functional interaction modules (31: *DCLRE1A*, *EXO1*, *KIAA0101*, *KIN*, *PCNA*, *POLD3*, *POLD2*; 35: *DKK3*, *FABP3*, *IRF1*, *AIM2*, *GBP1*, *GBP2*, *IRF2*) that are associated with prognosis of ovarian cancer patients. These network modules are related to DNA repair, replication, immune and cytokine mediated signaling pathways.

**Conclusions:**

The two 7-gene expression signatures may be accurate predictors of clinical outcome in patients with ovarian cancer and has the potential to develop new therapeutic strategies for ovarian cancer patients.

## Background

Ovarian cancer is the most common lethal gynecologic malignancy in women worldwide, with an estimated 22,280 newly diagnosed cases and approximately 14,240 deaths in 2016 in the United States [1]. Due to the lack of specific symptoms and effective screening tests, approximately 70 % of ovarian cancer patients have been in advanced-stage (stage III or IV) when they are firstly diagnosed, leading to the 5-year survival rate of less than 30 % [2]. By contrast, patients who are diagnosed with early-stage (stage I or II) have a 5-year survival rate of up to 70–90 % [2]. These data indicate the importance to identify the sensitive biomarkers to early distinguish the patients with different prognosis, aiming to determine optimal treatment strategies.

In the past years, remarkable achievements have been obtained in the investigation of prognostic markers for ovarian cancer. For instance, a 10-gene signature (*AEBP1*, *COL11A1*, *COL5A1*, *COL6A2*, *LOX*, *POSTN*, *SNAI2*, *THBS2*, *TIMP3*, and *VCAN*) has been validated to be associated with poor overall survival in patients with high-grade serous ovarian cancer [3]. The presence of a *BRCA1* or *BRCA2* mutation is associated with a better prognosis in patients with invasive ovarian cancer [4]. A recent study has found that suppression of *ABHD2* in OVCA420 cells increased phosphorylated p38 and ERK, platinum resistance, and side population cells, promoting a malignant phenotype and poor prognosis in serous ovarian cancer [5]. Furthermore, *CD73* enhances ovarian tumor cell growth and expression of antiapoptotic BCL-2 family members, indicating a role of *CD73* as a prognostic marker of patient survival in high-grade serous ovarian cancer [6]. Although the aforementioned genes have been shown to be correlated with the prognosis in ovarian cancer, their prognostic accuracy may be limited because the development of disease usually involves several genes and the interaction between them to form a complex pathway. Therefore, it is necessary to identify gene networks and pathways including multiple genes and their interactions, which can be achieved by Reactome functional interaction (FI) network construction as described previously [7, 8].

In the present study, we aimed to construct the Reactome FIs network to analyze the gene signatures that was significantly related to ovarian cancer patient survival based on gene expression profiling data extracted from The Cancer Genome Atlas (TCGA) database.

## Methods

As the paper did not involve any human or animal, the ethical approval was not required.

### Gene expression data

Two gene expression datasets with their corresponding clinical data (including survival status and time) for ovarian cancer samples were downloaded from TCGA database (https://tcga-data.nci.nih.gov/tcga). Data of one gene expression dataset were produced from the BI-HT-HG-U133A platform, in which 536 samples were included and 12042 genes were expressed in each sample (defined as BI). The other gene expression profiling from 559 ovarian cancer patients was produced from the UNC-AgilentG4502A-07-3 microarray platform, in which 17814 genes were included (defined as U3). These two datasets were randomly divided into training (BI) or testing sets (U3).

### Construction of Reactome FI network

The annotated FIs were extracted from five pathway databases, including Reactome [9], kyoto encyclopedia of genes and genomes (KEGG) [10], protein annotation through evolutionary relationship (Panther) [11], The Cancer Cell Map (http://cancer.cellmap.org/), and NCI Pathway Interaction Database (NCI-PID) [12]. The protein FIs were predicted by physical protein-protein interactions (PPIs) in human organisms (catalogued in the Biological General Repository for Interaction Datasets (BioGrid) [13], the Human Protein Reference Database (HPRD) [14] and IntACT [15]), model organisms (from IntAct [15] based on Ensembl Compara [16]), and protein domain–domain interactions (from PFam [17]). The naive Bayes classifier, a simple machine learning method [18], was used to score the probability that a protein pair-wise relationship reflects a functional pathway event, during which the annotated FIs were selected as positive training sets, whereas the predicted FIs were defined as negative training sets. Subsequently, the gene expression data of BI from the TCGA were mapped into the constructed Reactome FIs via co-expression relationships (calculated by Pearson correlation) to distribute the weight of each edge.

### Markov clustering (MCL)

The gene/protein correlations in the Reactome FI network were input into the Reactome FI Cytoscape plugin (MCL) [7] to generate a sub-network for a list of selected network modules based on module size (≥7) and average correlation (Pearson correlation coefficient ≥0.25). To control the size of network modules generated from the MCL clustering, the inflation coefficient was set as 5.0.

### Analysis of prognosis-related modules

The prognosis-related modules were further predicted based on the supervised principal components (superpc) [19] using the Superpc V1.05 software package under the programming environment R (http://statweb.stanford.edu/~tibs/superpc/). A module-based gene expression matrix was generated by using mean expression level of genes in each module across 536 ovarian cancer samples, and then underwent the superpc analysis. A 10-fold cross-validation curve was performed for estimating the best threshold. In addition, Cox proportional hazard (PH) model was also performed to correlate each module with survival data (*p* < 0.05), followed by Kaplan-Meier analysis to demonstrate the distinguishing ability of each module for survival.

### Gene Ontology (GO) functional and pathway annotations

The genes in prognosis-related modules were subjected to the GO and pathway enrichment analyses to identify their roles in ovarian cancer. GO and pathway functional annotations were conducted for the survival-associated genes using the Reactome FI plug-in of Cytoscape [20]. False discovery rate (FDR) < 0.05 was used for a threshold to assess the statistical significance.

## Results

### Data information

**Fig. 1:**
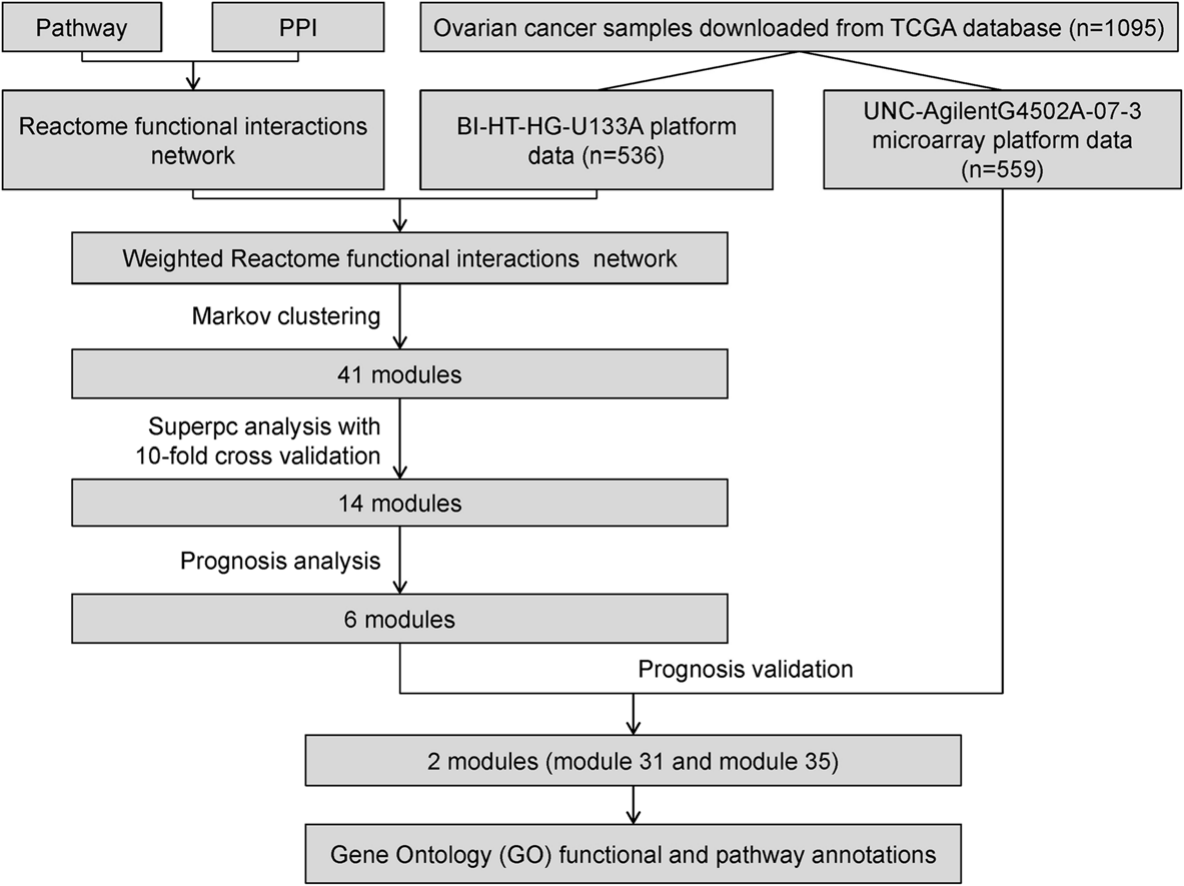
The flow chart of the whole analysis in this study

Two datasets [BI-HT-HG-U133A (BI), and UNC-AgilentG4502A-07-3(U2)] were obtained from TCGA. The BI dataset contained 536 samples, and expression data of 12042 genes were included in each sample. The U2 dataset contained 559 samples, and expression data of 17814 genes were included in each sample. In this study, BI was used as the training dataset, and U2 was used as the test dataset (Fig. 1).

### Analysis of the FI network and modules

Based on the BI dataset, a weighted FI network including 710 proteins and 9516 interactions were constructed. Subsequently, using MCL network clustering, a total of 41 modules were obtained, and the number of genes in each module ranged from 7 to 118. Furthermore, using the Superpc package with a threshold value of 0.73, 14 prognosis-related modules were identified from the 41 modules (Table 1). Afterwards, 6 significant modules (modules 6, 8, 20, 26, 31 and 35) with the *p*-value < 0.05 were identified from the 14 modules based on the Cox PH analysis (Table 2). These 6 modules were validated by the U2 dataset, and two modules (modules 31 and 35) were also significant in the U2 dataset. Thus, modules 31 and 35 were further analyzed.

**Table 1:** Superpc analysis for prognosis related modules according to 10-fold cross-validation method

Modules	Threshold	Cross-validation scores
1	0.050	9.667
6	0.119	9.096
8	0.187	7.953
12	0.255	7.146
14	0.324	8.551
19	0.392	7.371
20	0.460	6.903
25	0.529	8.649
26	0.597	8.848
27	0.665	9.558
28	0.734	10.394
31	0.802	10.177
35	0.870	7.547
36	0.939	7.414

**Table 2:** Cox proportional hazard analysis for prognosis related modules using the training (BI) and test datasets (U3)

Module	Size	BI		U3	
		Likelihood ratio	*p*-value	Likelihood ratio	*p*-value
26	9	9.41	0.002	0.32	0.574
20	10	6.91	0.009	3.58	0.058
31	7	7.43	0.006	4.45	0.035
35	7	6.19	0.013	6.25	0.012
6	22	5.96	0.015	2.57	0.109
8	21	6.22	0.013	0.67	0.414

### Analysis of modules 31 and 35

**Fig. 2:**
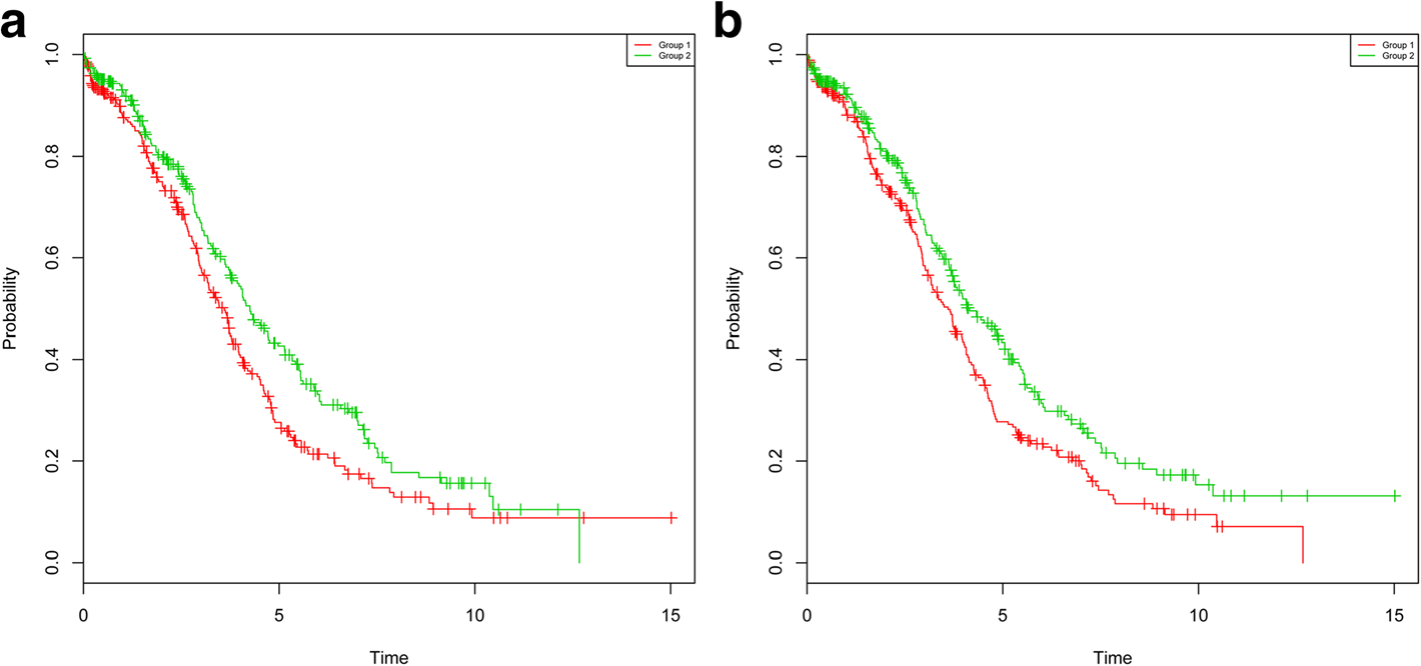
Kaplan-Meier survival plot for the module 31 (**a**) and 35 (**b**). All samples were divided into two groups based on the median value of gene expression in modules. The *green curve* is for samples having lower expression, while the *red curve* for samples having higher expression

**Fig. 3:**
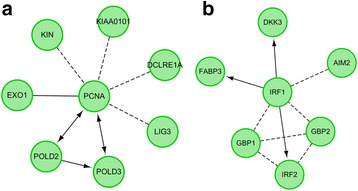
Genes and interaction relationship in the module 31 (**a**) and 35 (**b**). The *arrow* indicates the co-expression relationship and known pathway regulatory relationship; the *dotted line* indicates the newly predicted interaction; the *full line* indicates the common complex

A set of 7 genes (*DCLRE1A*, *EXO1*, *KIAA0101*, *KIN*, *PCNA*, *POLD3*, *POLD2*) were included in the module 31 (Fig. 2a), and 7 genes (*DKK3*, *FABP3*, *IRF1*, *AIM2*, *GBP1*, *GBP2*, *IRF2*) were included in the module 35 (Fig. 2b). Kaplan-Meier plot demonstrated that the gene expression in these two modules can significantly distinguish the patients with longer and shorter survivals (Fig. 3).

**Fig. 4:**
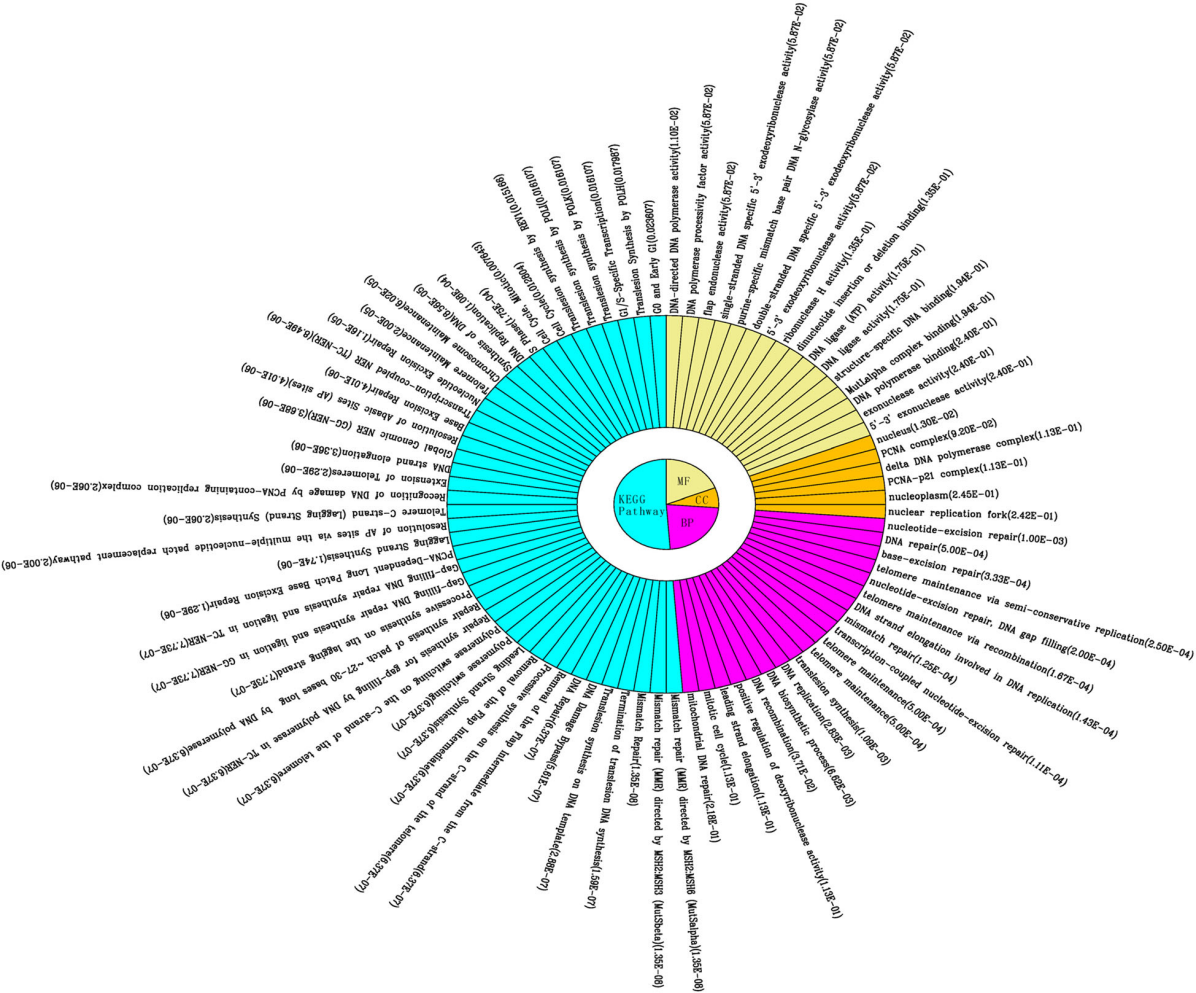
GO functional and KEGG pathway enrichment analyses of the genes in the module 31. MF, CC and BP are the three categories of the GO functional enrichment analysis. *GO* Gene Ontology, *KEGG* Kyoto Encyclopedia Of Genes And Genomes, *BP* biological process, *CC* cellular component, *MF* molecular function

**Fig. 5:**
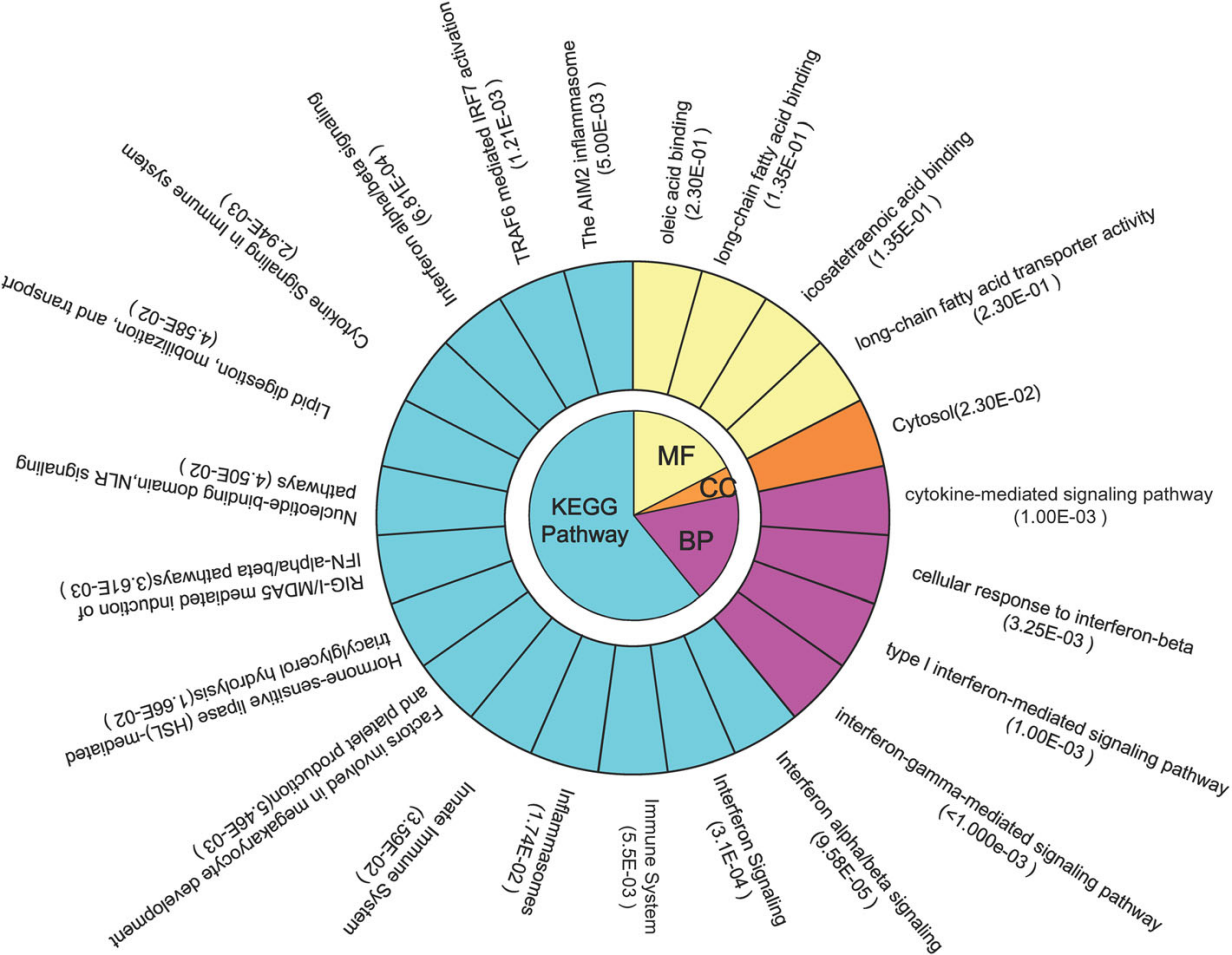
GO functional and KEGG pathway enrichment analyses of the genes in the module 35. MF, CC and BP are the three categories of the GO functional enrichment analysis. *GO* Gene Ontology, *KEGG* Kyoto Encyclopedia Of Genes And Genomes, *BP* biological process, *CC* cellular component, *MF* molecular function

To further investigate the biological functions of the genes in modules 31 and 35, GO and pathway annotations were performed. The genes in module 31 were mainly related to the functions of DNA repair, DNA replication and cell cycle (Fig. 4). The genes in module 35 were significantly associated with functions about immune and cytokine or interferon mediated signaling pathways (Fig. 5).

## Discussion

In this study, a total of 41 modules were obtained from the FI network based on the expression data in the BI dataset. Using MCL network clustering, superpc modeling and Cox PH analysis, two modules, modules 31 and 35, were identified to be significantly associated with prognosis of ovarian cancer patients. Seven genes were included in the two modules (31: *DCLRE1A*, *EXO1*, *KIAA0101*, *KIN*, *PCNA*, *POLD3*, *POLD2*; 35: *DKK3*, *FABP3*, *IRF1*, *AIM2*, *GBP1*, *GBP2*, *IRF2*). Furthermore, the genes in module 31 were related to DNA repair or replication, whereas the genes in module 35 were associated with immune and cytokine interferon mediated signaling pathways.

*DCLRE1*, also known as *SNM1A*, belongs to a member of a small gene family that is characterized by a metallo-β-lactamase fold and an appended β-CASP domain that together are proposed to function as a DNA endonuclease to participate in DNA inter-strand cross-link repair [21]. DNA cross-link repair is beneficial to maintain genomic stability and enables cells to survive DNA damage, contributing to less risk of tumorigenesis [22]. However, recent studies indicate that the high efficiency of DNA cross-link repair may also promote the excessive proliferation of cells, driving tumor initiation and progression [23–25]. Thus, down-regulation of DNA repair genes may be a promising target for anticancer therapy [26], which has been demonstrated by the study of Wu et al. [27]. Wu et al. have found that *DCLRE1A* is significantly decreased by bufalin, which promotes lung cancer apoptosis [27]. In addition, inhibition of DNA cross-link repair was also proved to reverse treatment resistance and improve the therapeutic efficacy [28].

*EXO1* encodes exonuclease and plays important roles in mismatch repair by resecting the damaged strand. Similar to *DCLRE1A*, *Exo1* is also shown to be higher expressed in tumor tissues than that in the normal tissues [29, 30]. A previous study has demonstrated that *FOXM1* facilitates DNA repair through regulating direct transcriptional target *EXO1* to protect ovarian cancer cells from cisplatin-mediated apoptosis, and attenuating *EXO1* expression by small interfering RNA augments the cisplatin sensitivity of ovarian cancer cells [31]. *POLD2* or *POLD3* are both the subunits of DNA polymerase delta that possesses both polymerase and 3′ to 5′ exonuclease activity and plays a critical role in DNA replication and repair [32]. *POLD2* was found to be increased in average 2.5- to almost 20-fold in moderately and poorly differentiated serous carcinomas of epithelial ovarian cancer, eventually leading to poor prognosis [33].

Furthermore, proliferating cell nuclear antigen (*PCNA*) is a ring-shaped homo-triomeric protein that functions as a necessary clamping platform to recruit numerous enzymes involved in DNA replication and repair, such as DNA polymerases, endonuclease, and DNA ligase, ultimately responsible for cell proliferation [34]. Therefore, *PCNA* is widely considered as a biomarker for cancer progression and prognosis. A recent study has found that *PCNA* was expressed in 52.2 % of gastric cancer patients, and positive expression of *PCNA* was significantly associated with poor 3-year disease-free survival (*p* = 0.035) [35]. *KIAA0101* is a 15-kDa protein that has a conserved motif to bind to *PCNA* via a yeast two-hybrid system and thus involved in the regulation of DNA repair and cell proliferation [36]. Similar to *PCNA*, overexpression of *KIAA0101* can promote growth and invasion of cancer cells [37] and predict poor prognosis in cancer patients [38, 39]. Collectively, these genes in the module 31 may play critical roles in the prognosis of ovarian cancer via regulation of DNA repair and cell proliferation.

In the module 35, 7 genes were included. Interferon regulatory factor 1 (*IRF1*) is a member of the interferon regulatory transcription factor (IRF) family, which can cause the inhibition of cell proliferation and stimulation of apoptosis [40]. *IRF2* is a functional antagonist of *IRF1* and may act as an oncogene, promoting the formation and progression of cancer [41]. A previous study has demonstrated that increased level of *IRF1* is associated with both increased progression-free and overall survival of patients with ovarian carcinoma, and *IRF1* is an independent predictor of platinum resistance and survival in high-grade serous ovarian carcinoma [42]. Furthermore, *IRF1* directly mediates the interferon-γ (IFN-γ)-induced apoptosis via the activation of caspase-1 gene expression in IFN-γ-sensitive ovarian cancer cells [43]. However, in a recent study of ovarian cancer, IRF-1 was identified to be up-regulated in ovarian cancer samples compared with healthy ovarian tissue although strong expression of IRF-1 predicted improved disease-free survival and overall survival [44]. This finding may be attributed to a compensation or adaptation mechanism. Further study indicated the *IRF1* seemed to play a key role in the transcriptional activation of interferon-inducible guanylate binding proteins (*GBP1* and *GBP2*) [45], which subsequently induces T-lymphocyte immune response against the cancer cell spreading and proliferation [46]. Therefore, *GBP1* and *GBP2* may be also tumor suppressor genes and associated with better prognosis [47].

*AIM2* is another human IFN-inducible protein, which forms the *AIM2* inflammasome with an adaptor protein ASC upon sensing foreign cytoplasmic double-stranded DNA [48]. The activated *AIM2* inflammasome in macrophages promotes the proteolytic cleavage and secretion of pro-inflammatory cytokines (IL-1β and IL-18) through the activation of caspase-1, leading to cell senescence, apoptosis and preventing cancer progression [49]. Thereby, *AMI2* may be also correlated with excellent prognosis [50, 51].

## Conclusion

Based on gene expression profiling data, two 7-gene functional interaction modules were identified to be likely associated with prognosis of ovarian cancer patients. These network modules were related to DNA repair, replication, immune and cytokine mediated signaling pathways. However, further experimental studies are required to confirm these genes in the modules.
